# Harnessing genomics in the battle against antimicrobial resistance and neglected tropical diseases

**DOI:** 10.1016/j.ebiom.2020.103178

**Published:** 2020-12-16

**Authors:** David A.B. Dance, Elizabeth M. Batty

**Affiliations:** a Lao-Oxford-Mahosot Hospital-Wellcome Trust Research Unit, Microbiology Laboratory, Mahosot Hospital, Lao People's Democratic Republic; b Centre for Tropical Medicine & Global Health, Old Road Campus, University of Oxford, United Kingdom; c Faculty of Infectious and Tropical Diseases, London School of Hygiene and Tropical Medicine, United Kingdom; d Mahidol Oxford Tropical Medicine Research Unit, Faculty of Tropical Medicine, Mahidol University, Thailand

Melioidosis is a severe and frequently fatal disease caused by an environmental bacterium, *Burkholderia pseudomallei*, that is widespread throughout the tropics. It is estimated to affect approximately 165,000 people every year and kill 89,000 of them [1]. Despite this, it remains little known, even in countries in which it is endemic, and the burden of infection is significantly underestimated by national surveillance systems [2,3]. It is so neglected that it does not even feature in official lists of Neglected Tropical Diseases (NTD) even though it is believed to cause loss of more disability-adjusted life years than many diseases formally recognised as NTDs [4].

In this issue of *EBioMedicine*, Madden and colleagues report the development of a tool, which they call ARDaP, for detecting antimicrobial resistance (AMR) determinants in next generation sequencing (NGS) data for *B. pseudomallei,* having found shortcomings with existing tools for this purpose [5]. They found that ARDaP accurately detected all the previously described SNP, indel, CNV, inversion, and gene loss AMR determinants in the *B. pseudomallei* isolates studied, and accurately predicted three previously undescribed determinants. In comparison, other commonly-used tools such as CARD, ResFinder and AMRFinderPlus failed to find any of the clinically-relevant variants in *B. pseudomallei*, and found only the intrinsic resistance genes present in all *B. pseudomallei* strains.

In many ways, *B. pseudomallei* was a strange choice of organism for this study, reflecting the prior research interests of the authors. Although it is a species that is intrinsically resistant to many antibiotics which has a large and highly plastic genome organised in two chromosomes, and exhibits frequent horizontal gene acquisition [6], probably reflecting its need to adapt to competition within its environmental niche, acquired AMR is not such a major problem amongst patients being treated for melioidosis as it is for many other infections [7]. The reason for this is probably two-fold. First, although resistance does occur in a minority of melioidosis patients during treatment, this is always a result of chromosomal mutations, and plasmid-mediated transferrable resistance, such as occurs amongst Enterobacterales for example, has never been reported [8]. Secondly, person-to-person spread of *B. pseudomallei* has rarely if ever been seen, and so even when resistance does develop it is an evolutionary dead end. In addition, whilst ARDaP may be an extremely efficient way of detecting AMR determinants in *B. pseudomallei*, it is unlikely to be applicable in the majority of clinical laboratories that serve the poor, rural populations in the developing world that are most likely to suffer from melioidosis, at least for the foreseeable future.

In fact, the most important aspect of the study of Madden et al. is the potential for this approach to be adapted for use with any bacterial species which, like *B. pseudomallei*, may not be included any datasets used to test AMR tools. There are many existing tools for determining AMR from sequencing data [9], but this study demonstrates that they are not all suitable for detecting resistance mechanisms in pathogens where resistance is caused by a wide spectrum of mutations. In addition, ARDaP is able to identify minor allele variants, enabling the prediction of AMR from samples where a single colony cannot be obtained, and to flag previously unknown potential AMR determinants for further investigation, a useful addition for species in which knowledge of AMR determinants is incomplete. While the components of this bioinformatic pipeline are all individually available, ARDaP combines them in an open-source package which produces a single user-friendly report, which will be invaluable for non-specialist bioinformaticians. However, accurate detection of *B. pseudomallei* resistance determinants required Madden et al. to develop a custom database for this species. To extend the use to other species will require substantial effort to curate catalogues of species-specific resistance variants, and to make those available to the scientific community, as has been done for other widely used AMR databases [10]. A further barrier to the adoption of NGS as a tool for use with pathogens predominantly identified in resource-limited settings is that these databases can only be as comprehensive as the number of phenotypically-characterised strains available permits. Detailed phenotyping and sequencing are not routinely performed in these settings, as can be seen from the limited number of strains available to Madden et al. despite the high burden of *B. pseudomallei* disease. Despite these limitations, ARDaP represents an important step towards incorporating AMR detection from NGS into routine clinical practice in the future.

## Contributors

The authors confirm sole responsibility for the conception and preparation of this invited Commentary.

## Declaration of Competing Interest

Dr. Dance reports personal fees from InBios, outside the submitted work; Dr. Batty has nothing to disclose.

## References

[1] Limmathurotsakul D., Golding N., Dance D.A.B. (2016). Predicted global distribution of *Burkholderia pseudomallei* and burden of melioidosis. Nature Microbiol.

[2] Chansrichavala P., Wongsuwan N., Suddee S. (2015). Public awareness of melioidosis in Thailand and potential use of video clips as educational tools. PLoS ONE [Electronic Resource].

[3] Hantrakun V., Kongyu S., Klaytong P. (2019). Clinical epidemiology of 7126 melioidosis patients in Thailand and the implications for a national notifiable diseases surveillance system. Open forum Infect.

[4] Birnie E., Virk H.S., Savelkoel J. (2019). Global burden of melioidosis in 2015: a systematic review and data synthesis. Lancet Infect Dis.

[5] Madden D.E., Webb J.R., Steinig E.J., Currie B.J., Price E.P., Sarovich D.S (2021). Taking the next-gen step: comprehensive antimicrobial resistance detection from *Burkholderia pseudomallei*. EBioMedicine.

[6] Holden M.T., Titball R.W., Peacock S.J. (2004). Genomic plasticity of the causative agent of melioidosis, *Burkholderia pseudomallei*. PNAS.

[7] Wuthiekanun V., Amornchai P., Saiprom N. (2011). Survey of antimicrobial resistance in clinical *Burkholderia pseudomallei* isolates over two decades in Northeast Thailand. Antimicrob Agents Chemother.

[8] Schweizer H.P. (2012). Mechanisms of antibiotic resistance in *Burkholderia pseudomallei*: implications for treatment of melioidosis. Future Microbiol.

[9] Su M., Satola S.W., Read T.D (2019). Genome-based prediction of bacterial antibiotic resistance. J Clin Microbiol.

[10] Feldgarden M., Brover V., Haft D.H. (2019). Validating the AMRFinder tool and resistance gene database by using antimicrobial resistance genotype-phenotype correlations in a collection of isolates. Antimicrob Agents Chemother.

